# Inelastic electron tunneling spectroscopy of difurylethene-based photochromic single-molecule junctions

**DOI:** 10.3762/bjnano.8.261

**Published:** 2017-12-06

**Authors:** Youngsang Kim, Safa G Bahoosh, Dmytro Sysoiev, Thomas Huhn, Fabian Pauly, Elke Scheer

**Affiliations:** 1 Chemical Sciences Division, Lawrence Berkeley National Laboratory, Berkeley, California 94720, United States; 2 Lam Research, Fremont, California 94538, United States; 3 Department of Physics, University of Konstanz, 78457 Konstanz, Germany; 4 Department of Chemistry, University of Konstanz, 78457 Konstanz, Germany; 5 Okinawa Institute of Science and Technology Graduate University, Onna-son, Okinawa 904-0395, Japan

**Keywords:** inelastic electron tunneling spectroscopy, molecular junction, photochromic, single molecule

## Abstract

Diarylethene-derived molecules alter their electronic structure upon transformation between the open and closed forms of the diarylethene core, when exposed to ultraviolet (UV) or visible light. This transformation results in a significant variation of electrical conductance and vibrational properties of corresponding molecular junctions. We report here a combined experimental and theoretical analysis of charge transport through diarylethene-derived single-molecule devices, which are created using the mechanically controlled break-junction technique. Inelastic electron tunneling (IET) spectroscopy measurements performed at 4.2 K are compared with first-principles calculations in the two distinct forms of diarylethenes connected to gold electrodes. The combined approach clearly demonstrates that the IET spectra of single-molecule junctions show specific vibrational features that can be used to identify different isomeric molecular states by transport experiments.

## Introduction

Molecular junctions hold promise for the realization of novel miniaturized electronic circuits [1–6] as well as for thermoelectric energy conversion devices [7–10]. Optoelectronic properties of several species of photochromic molecules in single-molecule junctions with gold electrodes have been investigated extensively to understand the mechanisms of charge transport and optical switching [11–16]. Among the photochromic molecules, diarylethene derivatives are particularly promising because of the negligible change of molecular length between the two isomers (i.e., open and closed forms) and the possibility for further chemical functionalization [13–14]. The isomerization upon illumination with appropriate wavelengths tunes the electronic structure of the molecules, including the energy of the highest occupied molecular orbital (HOMO) and the lowest unoccupied molecular orbital (LUMO) and their coupling to electrodes. This behavior results in a change of the charge transport properties of the corresponding molecular junctions, most prominently of the electrical conductance. Furthermore, the vibrational eigenmodes of both isomers will be different. To study the vibrational properties of molecular junctions, IET spectroscopy [4,17] was introduced, which is an electronic spectroscopy method applicable at low temperatures. IET measurements are capable of identifying the component molecules and are sensitive to the contact geometry, including molecular configurations and electrode–molecule couplings [18–20]. However, to understand the vibrational features of molecules measured by the IET spectroscopy technique, it is often essential to compare the experimental findings with theoretical predictions for the IET spectra. The reason is that IET spectroscopy is not subject to rigorous selection rules [21] in contrast for instance to optical vibrational spectroscopy methods such as infrared or Raman spectroscopy.

In this study, we investigate both elastic and inelastic charge transport through single-molecule junctions of a bis(furanylmethanthiol)ethene with a fluorinated cyclopentene bridging unit (C5F-ThM; for nomenclature see [22]). Experimentally measured electrical conductance and IET spectra are compared with first-principles calculations in open and closed forms of the photochromic molecule. For both isomeric molecular states, the features observed in the IET spectra are assigned with the help of computations that take the electron-vibration (EV) coupling into account.

## Results and Discussion

**Figure 1:**
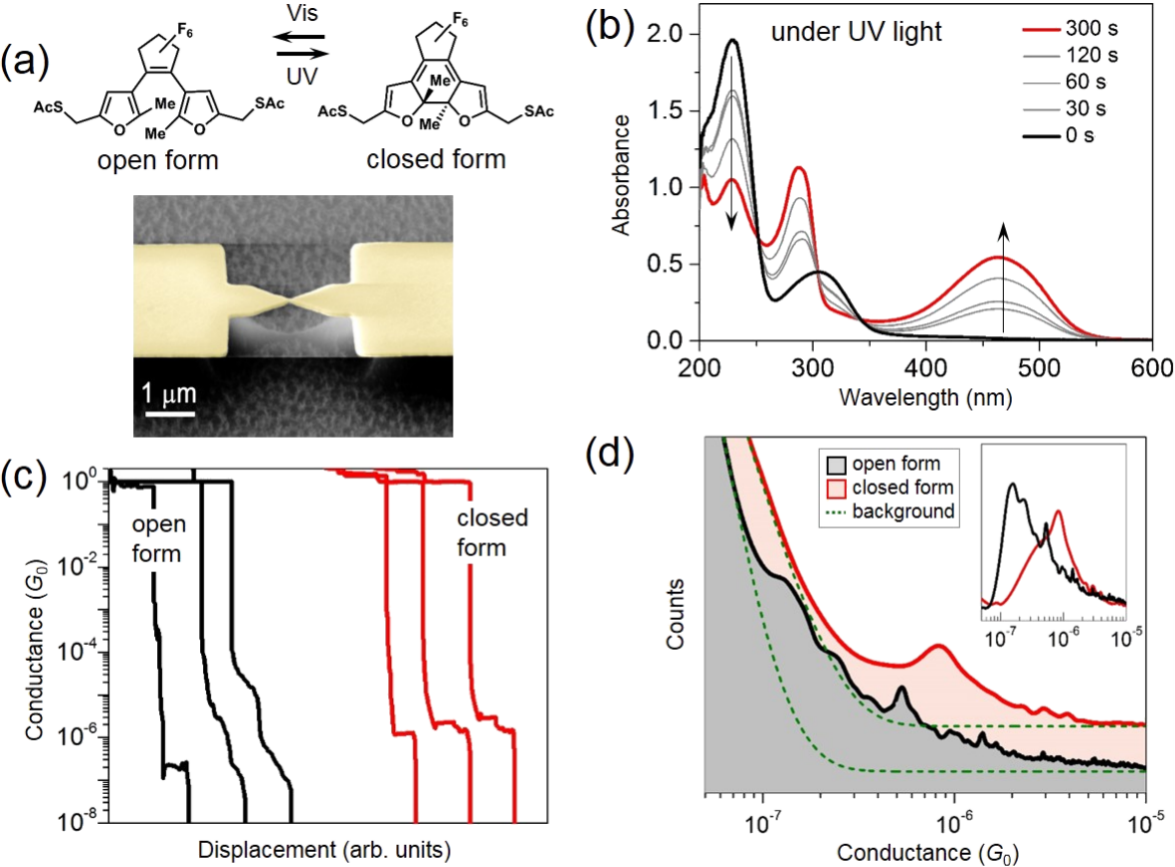
(a) Structures of the open and closed forms of difurylethene-thio-methyl (C5F-ThM) molecules, which switch states under the illumination with UV and visible light. Shown at the bottom is a scanning electron microscopy image of a MCBJ device. (b) Variation of UV–visible absorption spectra under UV light irradiation (wavelength of 313 nm with an intensity of ca. 0.15 mW/cm^−2^) as a function of illumination time. The black curve corresponds to the open form, the red one to the closed form. (c) Conductance traces recorded while breaking C5F-ThM molecular junctions, containing the open form (black) and the closed form (red). Curves are shifted along the displacement axis for clarity. (d) Conductance histograms constructed from around 1000 curves of breaking and forming processes. The lowest conductances for the open and closed forms are (1.4 ± 1.0) × 10^−7^ *G*_0_ and (8.3 ± 4.5) × 10^−7^ *G*_0_, respectively. Histogram curves of both forms (black and red lines) are displaced along the counts axis for better visibility. The inset shows the corresponding conductance histograms after subtracting the exponential backgrounds indicated by green dashed lines in the main panel.

The C5F-ThM photochromic molecule, used in this work, is illustrated in Figure 1a with its reaction schematics, when exposed to ultraviolet or visible light. The synthesis method was presented previously [22]. Upon illumination with light of suitable wavelength, the central carbon–carbon bond of the difurylethene switching core is either formed or broken, resulting in a change of the molecular orbitals. As can be seen in Figure 1b, we measured the absorption spectra of C5F-ThM molecules, while illuminating with UV light (313 nm) for different illumination times. The absorption band of the closed form extends to a higher wavelength (around 550 nm) than those of the open form (around 380 nm), suggesting that the closed form has a smaller HOMO–LUMO gap. The closed form exhibits delocalized orbitals, leading to a π-conjugated current path across the molecule. In the open form, the conjugated system is split into two tunnel-coupled halves. The molecules are switched to either the open or the closed form in ethanol and are subsequently assembled on gold-patterned MCBJ samples [14]. The acetyl (COCH_3_, denoted as Ac) groups are cleaved off by adding a droplet of ammonium hydroxide (NH_4_OH), resulting in a thiol (SH) group, before assembly on the metal surfaces of the sample. The MCBJ sample preparation [14] is as follows. Polyimide is spin-coated to be 2 μm in thickness on a softly polished bronze wafer (200 μm in thickness, CuSn_6_), and then the polyimide on the wafer is annealed for 6 hours at 430 °C in vacuum (10^−5^ mbar). Subsequently, the electron beam lithography is performed with a double layer of electron beam resists (copolymer/PMMA). After developing the resists, Au of around 70 nm in thickness is deposited, the electron-beam mask is lifted off in warm acetone, and then the polyimide layer is partially etched away (thickness reduction of ca. 700 nm) by employing O_2_ plasma in a reactive ion etcher to form a freestanding bridge, as shown in Figure 1a.

After mounting the MCBJ sample, which is covered with C5F-ThM molecules either in the open or the closed form, charge transport measurements were performed at low temperature (4.2 K) in a custom-made vacuum insert equipped with a MCBJ system [14–15,23]. The sample space of the cryostat is evacuated to 10^−5^ mbar, and then a small amount of He gas is injected as exchange gas. After cooling down the cryostat, the breaking process of the sample is carried out by the breaking mechanics, controlled by a DC motor and a differential screw. The low-bias conductance is measured by a sub-femtoamp source-meter (Keithley 6430) operating with an automatic variable gain pre-amplifier, while controlling the nanogap distance and thereby breaking and forming Au–Au contacts. In order to measure IET spectra, a DC bias, added to an AC modulation of 8 mV (root mean square) at a frequency of 317 Hz, is swept at a 1 mV/s rate using a low-noise DC source (Yokogawa 7651). Finally, the signals are amplified by a low-noise current amplifier (Ithaco 1211) and recorded with a lock-in amplifier (SRS 830).

When stretching an Au nanobridge, a single-atom contact of Au forms with high probability. The single-atom contact is signaled by an electrical conductance *G* close to 1 *G*_0_, where *G*_0_ = 2*e*^2^/*h* is the quantum of conductance. As shown in Figure 1c, upon further stretching of the junction the atomic contact breaks, and molecules can be trapped between the separated electrodes showing molecular conductance plateaus. The plateau with the lowest conductance indicates a single-molecule junction, before also the metal–molecule–metal contact breaks, as indicated by a sudden drop of the conductance to below 10^−8^ *G*_0_. This breaking process is reversed by closing the junction until Au–Au contacts form again. The breaking and forming processes are repeated roughly 1000 times to find the most probable single-molecule conductance. We note that the plateaus are well developed with a rather constant conductance upon stretching in the closed form, while they are shallow and declined in the open form. We attribute this difference to the flexible structure in the open form that enables a larger variation of contact geometries, resulting in a larger variation of conductance values [14,24–25].

The statistical conductance histogram is shown in Figure 1d. For both forms there is only one well developed maximum, each superimposed on a background of increasing height for decreasing *G* values as typical for molecular junctions [1]. However, for the open form two features are discernable at higher conductance values. From the examples of conductance traces shown in Figure 1c it becomes clear that multiple steps occur within the individual opening processes. These multiple steps might originate from multi-molecule contacts as well as from a single-molecule junction that adopts different configurations upon stretching. We assume that the most elongated junctions with the lowest conductance values correspond to the molecule being completely stretched and suspended between the electrodes, i.e., being connected to gold through the sulfur binding groups. Hence, the most probable conductance is found to be (1.4 ± 1.0) × 10^−7^ *G*_0_ and (8.3 ± 4.5) × 10^−7^ *G*_0_ for the open and closed form, respectively, based on a Lorentzian fit [14]. The conductance peaks are better visible, when an exponential background is subtracted, as shown in the inset of Figure 1d. The peak positions are slightly modified by this procedure, but remain in the given error margins. From the most probable conductance values we derive a conductance switching ratio between the open and closed form of 5.9 ± 5.3 times using error propagation to determine the error for the switching ratio. The lower conductance for the open form is expected due to the breaking of π-conjugation in the C5F-ThM molecules [14]. The overall magnitude of the conductance of both the open and the closed form is rather small. This can be attributed to the presence of sp^3^ hybridized methylene groups, isolating the π-system and the thiol anchor groups. These act as efficient tunneling barriers for the electronic transport. Furthermore, the relatively small difference in conductance of the open and the closed form is understandable from the presence of these alkyl tunneling barriers that limit the total conductance in the closed form and in a simplistic picture might be considered as series resistances. Yet, the variation of contact geometries must be taken into account. From the measurements at room temperature on difurylethenes with more complex [24] but conjugated side chains, it was concluded that the open form may adapt more easily to the interelectrode gap distance, such that its conductance is optimized, while in the closed form the junctions might be distorted leading to a smaller conductance than in an ideal geometry. As a result, the conductance ratios between the open and the closed forms would be relatively small.

**Figure 2:**
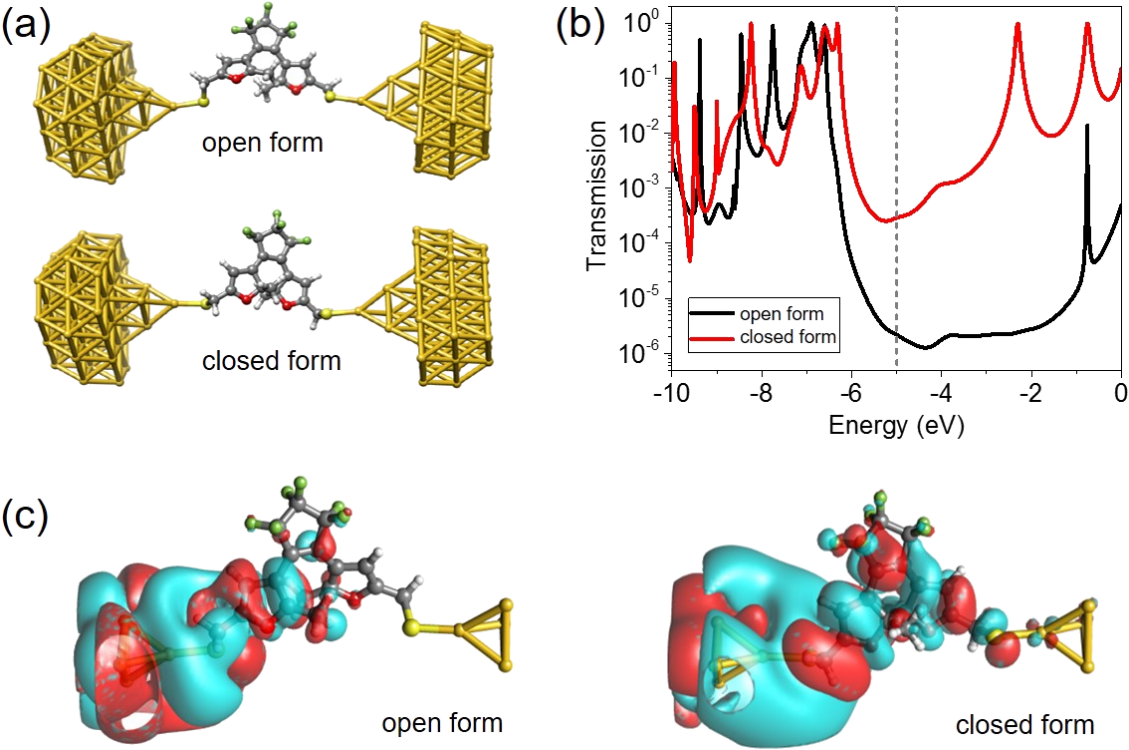
(a) The geometric structure used in the DFT calculations is displayed for the open and closed forms of Au-C5F-ThM-Au junctions. (b) The computed transmission curves for the open (black) and the closed form (red). (c) The wavefunction of the dominant transmission eigenchannel for electrons coming in from the left side for the open (left) and the closed form (right). Red and turquois indicate different signs of the wavefunction.

For a better understanding, we performed computations based on density functional theory (DFT) for charge transport through C5F-ThM molecular junctions. DFT, as implemented in the TURBOMOLE software package [26], was employed for the calculations using the exchange-correlation functional PBE [27–30] and the def-SV(P) [31–32] basis set, which is of split-valence quality with polarization functions on all non-hydrogen atoms [20]. To model charge transport through the junctions, either the open or closed form of the C5F-ThM molecule was connected to two Au clusters of 53 atoms to the left and the right side of the molecule, as shown in Figure 2a. The clusters resemble ideal Au <111> pyramids and represent the tips of the semi-infinite Au electrodes. In our case, the <111> direction coincides with the transport direction. There are various possibilities to bind the molecule to both electrodes [33]. In this work, the so-called “top position” is assumed, where each S linker atom of the molecule is connected to the topmost Au atom at the tip of the Au pyramids at both sides via a single covalent bond. We follow [33] to construct the junction geometries. First, we connect the molecule to one electrode, optimize the structure and then add the other electrode symmetrically. After connecting the molecule to the gold pyramids, those two gold layers that are closest to the molecule on each side are relaxed, while the other parts of the gold pyramids are kept fixed. This leads to approximate equilibrium geometries of the C5F-ThM single-molecule junctions with regard to the separation between the Au electrodes.

Having determined the junction geometries, we computed the elastic transmission functions of the single-molecule junctions for the open and closed molecular forms, as shown in Figure 2b. Since it is known that DFT with semilocal exchange-correlation functionals, as used here, tends to underestimate the HOMO–LUMO gaps of molecules and does not capture nonlocal surface polarization effects that are essential for an accurate description of metal–molecule level alignments, we use here the so-called DFT+Σ method [34–35]. By adding a self-energy correction, the DFT+Σ approach aims at improving the DFT-based energy levels of the molecules and has been reported to lead to a better agreement between experimental and theoretical conductance values [34]. Details regarding our DFT+Σ implementation can be found in [34,36]. In Figure 2b, the differences of the transmission functions of the two forms are clearly visible, and the transmission at the Fermi energy τ(*E*_F_) is found to be 2.17 × 10^−6^ and 2.9 × 10^−4^ for the open and the closed form, respectively. Further, the computed transmission functions indicate that the transport is strongly off-resonant and that the HOMO energy is closer to the Fermi energy than the LUMO level, as typical for sulfur anchoring groups [37].

In Figure 2c, we show the wavefunctions of the dominant eigenchannel at *E*_F_ for the closed and open forms for electrons that enter from the left side. The electric transport mainly proceeds through the π-electron system of C5F-ThM in both cases, as is visible from the different signs of the wavefunctions at the two sides of planes through the pentagonal carbon-based rings. For the closed form, the eigenchannel extends through the whole molecule from the left to the right. However, the π–π coupling between the rings is suppressed for the open form. In this case, the molecular orbitals localize on the left and right side. The incoming electron waves from the left lead couple through the sulfur atom into the π-electron system of the left side, but in the middle of the molecule at the core unit responsible for switching they are back-reflected. This largely suppresses the transmission, showing up in a low amplitude of the wavefunction that arrives on the right electrode.

Let us now discuss in more detail the results of Figure 2. For the isolated C5F-ThM molecules with SH termination, we find HOMO–LUMO gaps of 1.3 and 2.8 eV in the closed and open forms, respectively. They are corrected to 5.2 and 6.7 eV, if we use the DeltaSCF method [38]. As visible also from Figure 2b, the gaps are reduced to 4.2 and 5.8 eV in the molecular junctions due to image charge effects by the DFT+Σ approach. The consistently larger gap for the open form as compared to the closed one is in agreement with the conclusions from the absorption spectroscopy in Figure 1b.

Considering the shape of the transmission functions in both molecular forms in Figure 2b, it is clear that τ(*E*_F_) cannot simply be described by approximating the HOMO resonance with a Lorentzian, as assumed in the single-level model [37]. This is due to the contribution of other orbitals, involving mainly the sulfur anchors that lead to the faint bump at around 1.1 eV above the Fermi energy in both forms. The HOMO energy of the closed form is positioned around 1.3 eV away from *E*_F_, while this difference amounts to 1.6 eV in the open state. These values are both larger than the experimentally determined values of *E*_0_ of 0.54 ± 0.11 eV for the closed form and 0.86 ± 0.14 eV for the open form. They were extracted previously [14] from the analysis of current–voltage characteristics based on the two-parameter single-level model, where *E*_0_ specifies the position of an effective level with regard to the Fermi energy and Γ is the level broadening. Although the *E*_0_ values in the experiments are clearly smaller than the theoretically predicted differences of the closest molecular resonance, the HOMO level, to the Fermi energy, the trend of a decreasing *E*_0_ for the transition from the open to the closed state is well reproduced. A similar comparison can be made for the lifetime broadening Γ. It was determined to be (6.3 ± 1.2) × 10^−4^ eV for the closed form and (4.0 ± 1.0) × 10^−4^ eV for the open state. An analysis of the width of the computed HOMO resonance yields around 7 × 10^−3^ eV and 10^−3^ eV, respectively. Again the larger broadening of the HOMO level in the closed form as compared to the open one in the theory is in line with the increased effective level broadening Γ, determined from the current−voltage measurements.

Further discrepancies arise, if we quantitatively compare experimentally measured and theoretically predicted conductances. As stated above (see Figure 2), the predictions for the zero-temperature linear-response conductance of the closed and open forms are 2.9 × 10^−4^ *G*_0_ and 2.17 × 10^−6^ *G*_0_, respectively. The values for the most probable single-molecule conductance, as extracted from the conductance histograms, are (8.3 ± 4.5) × 10^−7^ *G*_0_ and (1.4 ± 1.0) × 10^−7^ *G*_0_ instead (see Figure 1). The conductance values obtained from the computations are hence larger than those deduced from the experiments by a factor of around 16 for the open and 349 for the closed form. Such deviations can appear, because the precise contact geometry in the experiments may be different than assumed in the computations. As argued for molecules of the same class measured at room temperature [24], it is reasonable to assume that geometrical constraints may affect the rigid closed form more than the flexible open form, thereby suppressing the conductance of the closed form more efficiently. Another explanation would be uncertainties with regard to level positions in our DFT-based procedure. In pure DFT calculations, i.e., without the corrections of the DFT+Σ method, we find that the HOMO level of the closed form is positioned right at the Fermi energy, as typically the case for large conjugated molecules, while for the open form the HOMO resonance is located around 0.2 eV below *E*_F_. The pinning of the HOMO level at *E*_F_ for the closed form may lead to enhanced uncertainties with regard to the description of the level alignment in this molecular state with its extended conjugated electron system as compared to the open form with its interrupted π system. Although the DFT+Σ method applied here is supposed to reduce level-alignment and band-gap errors, they might not be completely lifted in the closed form. Another important uncertainty, this time on the experimental side, stems from the interpretation of particular peaks in the conductance histograms. We consistently assign the peak with the lowest conductance as originating from the single-molecule junction. However, the fact that several peaks are observed may also indicate that single-molecule junctions with different conductance values, depending on the geometry, exist.

**Figure 3:**
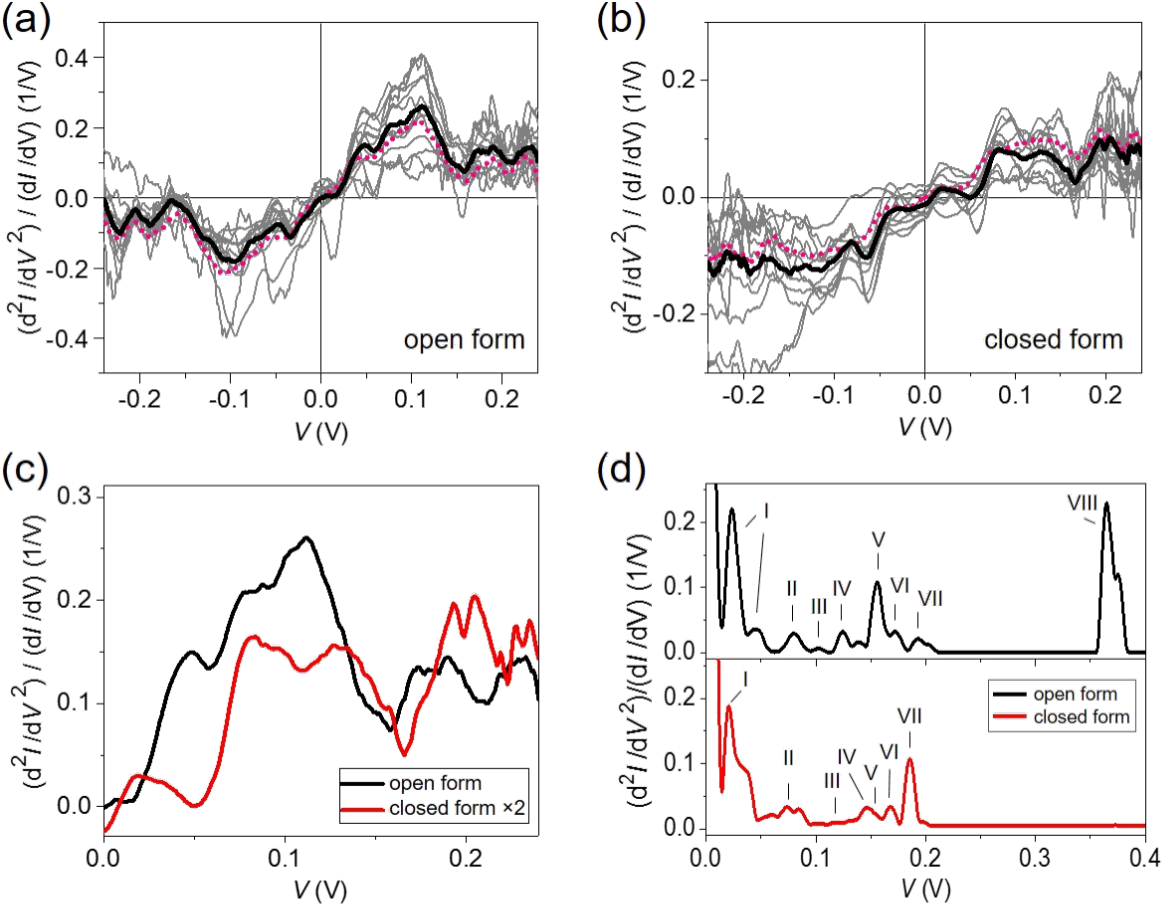
Experimentally measured IET spectra (gray) are displayed for (a) the open and (b) the closed form. For each form twelve curves were averaged (solid black line) and the average was symmetrized (pink-dotted line), using the function *y* = [*f*(*x*) − *f*(−*x*)]/2. (c) Averaged and symmetrized IET spectra for the open (black) and the closed (red) form, displayed only in the positive bias range. The IET spectrum of the closed form is magnified by a factor of 2. (d) Theoretically computed IET spectra for the open (upper panel, black) and closed (lower panel, red) form. Each mode, indicated with Roman numerals, is listed in Table 1.

Subsequently, we measured IET spectra for the two different forms of C5F-ThM molecular junctions and compared them with computed IET spectra, as shown in Figure 3. The excitations of molecular vibrations appear as peaks in the second derivative of current–voltage characteristics (in the positive bias regime) and can be detected using the lock-in technique [15,19–20]. In this study, the second derivative (d^2^*I*/d*V*^2^) is measured simultaneously with the differential conductance (d*I*/d*V*) by means of two lock-in amplifiers. The second derivative is normalized with d*I*/d*V* to compensate for the conductance change. Thus the IET spectroscopy amplitude is defined as (d^2^*I*/d*V*^2^)/(d*I*/d*V*) [19]. IET measurements were performed, when the samples exhibit single-molecule junctions, as signaled by conductance values in the lowest conductance plateau. As can be seen in Figure 3a and 3b, twelve IET spectra were measured on open and closed forms, respectively, and the curves were averaged to yield the black-solid lines. This averaging procedure is performed, since the recording of IET spectra for the low-conducting C5F-ThM is very challenging. We note also that the individual spectra have been recorded for different breaking events and thus correspond to different contact and electrode geometries. Finally, the averaged IET spectra are symmetrized using the function *y* = [*f*(*x*) − *f*(−*x*)]/2, since the vibronic excitations are expected to show antisymmetric features in the IET spectra with respect to bias reversal [15]. This results in the pink-dotted curves. The good agreement between the averaged and the symmetrized data shows that the spectra for both forms are mainly antisymmetric. This corroborates that the observed features are indeed caused by inelastic excitations, because other phenomena like, e.g., conductance fluctuations would give rise to no particular symmetry [39]. The pronounced peaks and dips in the IET spectra at positive and negative bias are caused by inherent molecular vibrations. In Figure 3c, the averaged IET spectra of both open and closed forms are presented for positive bias. Vibrational peaks are especially noticeable between 100 and 250 mV, which is considered to be the “fingerprint regime” of molecular junctions. At lower bias, contributions by the electrodes may dominate. In the closed form, peaks appear blue shifted compared to the open form. Considering the scaling factor of 2 for the closed form in Figure 1c, the amplitudes of the modes in the open form are higher than in the closed one at all energies.

To elucidate the origin of the different IET spectra in the two forms, we have computed the inelastic transport of the optimized geometries shown in Figure 2a. For the theoretical description of the IET spectra, the vibrational modes and the EV coupling constants were evaluated using DFT [40]. Inelastic charge transport is then determined in the experimentally relevant limit of weak EV coupling, as described in [40] and [41], by treating inelastic interactions due to the EV coupling at the level of the so-called lowest-order expansion (LOE) [40]. Instead of using the DFT effective single-particle Hamiltonian directly, we replace it with the one obtained from the DFT+Σ procedure to better describe quasiparticle level positions in the molecular junctions and to reproduce the elastic transport of Figure 2 in the regime of vanishing EV couplings [42]. In the calculations of the theoretical IET spectra we assume the wide-band limit [43], a temperature of 4.2 K and a vibrational broadening of 1 meV. The latter parameter takes into account that IET signals acquire an intrinsic line-width due to a finite temperature and finite lifetime of the vibrational modes. Furthermore, we broaden the spectra by the experimental AC excitation of 8 mV. The computed IET spectra for both forms are presented in Figure 3d, and they are seen to be distinct in both forms. We note that typically several vibrational modes contribute to a peak in the IET signals, and an approximate character is assigned to those vibrational modes that are responsible for the peaks in the spectra, as shown in Table 1. The most pronounced difference between the IET spectra is predicted at around 365 mV, where the intensity of the C–H stretching mode (mode VIII in Table 1) for the open form vanishes for the closed form. This voltage range was, however, not explored in the experiments due to the enhanced noise in the higher bias regime. In between 100 and 250 mV the vibrational modes of these two isomers appear at slightly different energies, but the major difference are the intensities of the modes. In the closed form, the C–H bending (III) and C–F stretching (IV) modes are blue-shifted, whereas the other modes are slightly red-shifted. While the intensity of the C–O stretching mode (V) is high in the open form, it is small in the closed form. The opposite holds for the C–C stretching mode (VII), which is stronger in the closed than in the open form. A detailed analysis reveals that the modes involving the atoms of the central ring that opens and closes upon photoreaction lie around 190 meV. It can be expected that the IET peaks related to these modes are reduced in amplitude in the open form of C5F-ThM.

**Table 1:** Approximate vibrational mode assignment for the theoretically computed IET spectra of Au-C5F-ThM-Au molecular junctions.

Mode	Description	Peak position (mV)	
		open form	closed form

I: ν(Au–S)	Au–S stretching	24; 45	21
II: ν(C–S)	C–S stretching	80	78
III: γ(C–H)	C–H bending	102	119
IV: ν(C–F)	C–F stretching	125	146
V: ν(C–O)	C–O stretching	156	154
VI: γ(C–H)	C–H bending	173	168
VII: ν(C–C)	C–C stretching	194	185
VIII: ν(C–H)	C–H stretching	365	–

The comparison of the theoretically computed IET spectra with the measured ones is not easy due to the rather broad peak features found in the experiments. We want to stress again the challenge to record these spectra for such low-conducting molecules, requiring an averaging procedure over several spectra, as explained above. Both experimental and theoretical results show, however, that IET spectra can distinguish between the different states of the functional molecular switching core through the different vibrational properties of either the open or closed form.

## Conclusion

We have studied the elastic and inelastic charge transport behavior of difurylethene-derived single-molecule junctions at low temperatures both experimentally and theoretically. We have found that the HOMO is much closer to the Fermi energy than the LUMO, but the energy-dependent transmission still suggests that the single-level toy model is not straightforwardly applicable. Based on the position of the HOMO level and its broadening in the transport calculations, we have nevertheless argued that experimental trends of an effective level that is better aligned with the metal’s Fermi energy and better coupled to the electronic states of the electrodes, when going from the open to the closed molecular state, are consistent with theoretical expectations. These trends naturally result in a higher electrical conductance of the closed form, as observed both experimentally and theoretically. In this context, we have also seen that the HOMO–LUMO gap in the closed form is smaller than in the open one. Quantitatively, theory and experiment differ by one order of magnitude in the conductance of the open form but two orders in the closed one, which requires further investigations. Points to examine are the accuracy of theoretically described level alignments for molecules with extended π-electron systems and ideally a representative ensemble of junction geometries as well as the possibility of the existence of several single-molecule junction geometries with differing conductance. Finally, we have performed an IET spectroscopy study and have shown that different IET spectra are obtained experimentally and theoretically for open and closed forms. IET spectroscopy can hence be used to distinguish between different molecular “on” and “off” states by means of their vibrational features. These findings advance the development of functional molecular electronics and prove at the same time that IET spectroscopy is a valuable technique to probe the isomeric states of individual molecules.
